# Effect of Exercise Training on Quality of Life after Colorectal and Lung Cancer Surgery: A Meta-Analysis

**DOI:** 10.3390/cancers13194975

**Published:** 2021-10-03

**Authors:** Pedro Machado, Sara Pimenta, Bárbara Oliveiros, José Pedro Ferreira, Raul A. Martins, Joana Cruz

**Affiliations:** 1 Center for Innovative Care and Health Technology (ciTechCare), School of Health Sciences of the Polytechnic of Leiria, 2411-901 Leiria, Portugal; 5160250@my.ipleiria.pt (S.P.); joana.cruz@ipleiria.pt (J.C.); 2 University of Coimbra, Research Unit for Sport and Physical Activity (CIDAF, UID/PTD/04213/2019), Faculty of Sport Sciences and Physical Education, 3040-248 Coimbra, Portugal; jpferreira@fcdef.uc.pt (J.P.F.); raulmartins@uc.pt (R.A.M.); 3 Laboratory of Biostatistics and Medical Informatics (LBIM), Faculty of Medicine, University of Coimbra, 3000-548 Coimbra, Portugal; boliveiros@fmed.uc.pt; 4 Faculty of Medicine, Coimbra Institute for Clinical and Biomedical Research (iCBR), University of Coimbra, 3000-548 Coimbra, Portugal; 5 Institute for Biomedical Imaging and Translational Research (CIBIT), University of Coimbra, 3000-548 Coimbra, Portugal

**Keywords:** aerobic exercise, colorectal cancer, fatigue, lung cancer, meta-analysis, prehabilitation, quality of life, rehabilitation, resistance exercise, surgical oncology

## Abstract

**Simple Summary:**

Surgery is the treatment modality associated with better long-term survival in patients diagnosed with lung cancer and colorectal cancer. However, as a result of surgery, patients experience a substantial decline in health-related quality of life (HRQoL) and increased fatigue symptoms. The purpose of this systematic review was to investigate the effect of pre- and/or postoperative exercise training on HRQoL and fatigue after surgical resection for lung and colorectal cancer. Our results showed that exercise training interventions improve the physical domain of HRQoL and reduce fatigue levels after lung cancer surgery, supporting its use to optimize patients’ recovery. No benefits were found on HRQoL and fatigue after colorectal cancer surgery.

**Abstract:**

Surgical treatment affects health-related quality of life (HRQoL) and increases fatigue symptoms in patients with lung cancer (LC) and colorectal cancer (CRC). We aimed to systematically review the effect of exercise training on HRQoL and fatigue after LC and CRC surgery. Randomized controlled trials published before 21 March 2021, were searched in PubMed, Scopus, Web of Science, SPORTDiscus and PEDro. Eligible trials compared the effect of exercise interventions initiated preoperatively or in the first 3 months after surgery versus usual care on postoperative HRQoL and fatigue. Standardized mean differences (SMD) were pooled using random-effects models. Twelve studies with a total of 777 patients were included. In LC patients (10 studies, n = 651), exercise training in general led to a moderate improvement in the physical domain of HRQoL (0.68: 95% CI: [0.47; 0.89]) and a small reduction in fatigue levels after surgery (SMD = 0.28: 95% CI: [0.02; 0.53]), while no effects were found in other HRQoL domains. In CRC (two studies, *n* = 126), exercise training showed no effects on HRQoL and fatigue after surgery. Exercise training is an effective intervention to improve physical function and fatigue after LC surgery. Further studies are necessary to clarify the effects of exercise on HRQoL and fatigue after CRC surgery.

## 1. Introduction

The epidemiologic relevance of cancer is growing worldwide, with an incidence of 19.3 million new cancer cases and almost 10.0 million cancer deaths in 2020 [1].

Lung cancer (LC) and colorectal cancer (CRC) account for over 21% of global cancer incidence and are the leading causes of cancer death, with numbers in 2020 reaching 1.8 million deaths worldwide by LC (18%) and almost 1 million by CRC (9.4%) [1].

In the early stages of these tumors, surgical resection is the primary treatment modality, and the intervention associated with better long-term prognosis [2,3]. However, as a result of surgical resection, there is a significant deterioration in patients’ health-related quality of life (HRQoL) [4,5,6,7,8,9].

A study conducted after lobectomy found that 100% of the patients reported being concerned about the limitations in their physical function and 96% about the levels of fatigue and pain [10]. Although, in general, HRQoL progressively returns to preoperative levels between 3 to 6 months after LC surgery, it has been shown that domains like physical function, fatigue and dyspnea persist significantly worse than at the preoperative phase for at least 1 to 2 years after surgery [4,7,9,11,12,13], even in patients selected for LC resection by video-assisted thoracoscopic surgery (VATS) approaches [4].

With respect to CRC, findings are similar, with HRQoL dropping significantly below the preoperative values one month following the surgery and fatigue being identified as the most troublesome problem 1 and 5 weeks postoperatively [6]. Regarding long-term recovery, approximately 40% of the patients reported a worse HRQoL 6 months after surgery and about one-third did not return to pre-surgery levels five years after treatment [14].

Therefore, when aiming to improve patient-centered care, where the individual’s perspective is emphasized, it becomes important to find interventions that could enhance HRQoL and reduce fatigue severity following CRC and LC resections [4,14].

One intervention that has been consistently shown to improve cancer patients HRQoL is exercise training [15,16,17]. The most recent international consensus about exercise prescription in oncology found strong evidence of the therapeutic benefits of exercise programs on cancer-related fatigue, anxiety, depressive symptoms and physical function, during and after cancer treatment [15]. The expert panel found that the majority of these cancer health-related outcomes are improved by doing moderate intensity aerobic exercise thrice-weekly for 30 min, and that there is also evidence of benefit with a twice-weekly resistance exercise program [15].

In the context of surgery for CRC and LC, exercise training has been implemented either as a prehabilitation intervention aiming to optimize the preparation of patients for tumor resection, or as a rehabilitation strategy to improve postoperative recovery [18]. The results of clinical trials support the benefits of perioperative exercise training on aerobic capacity and muscle mass/strength, components of physical fitness associated with better HRQoL in CRC and LC patients [19,20,21,22]. A high intensity interval training program before rectal cancer surgery was found to reverse the decline in aerobic capacity caused by neoadjuvant chemoradiotherapy [23] and programs involving aerobic plus resistance exercise have been shown to prevent the decline in muscle strength after lobectomy [24] and attenuate the skeletal muscle catabolism induced by CRC surgery [25,26]. Moreover, previous systematic reviews concluded that CRC and LC patients who underwent pre or postoperative exercise training had better exercise capacity [27,28,29,30], which is an important determinant of postoperative prognosis [31,32,33]. Despite that, there is no clear evidence that this improvement in exercise capacity translates into a better HRQoL following CRC and LC resections [28,34].

HRQoL is a multidimensional construct encompassing patients’ perceptions of domains such as physical, emotional, social, and cognitive functions [35], and these perceptions are influenced not only by exercise capacity [20], but also by clinical factors such as the administration of adjuvant chemotherapy [36,37], specific symptoms such as fatigue and pain [38,39,40], the extent of surgical resection [12,13,40,41], and psychosocial determinants such as anxiety, depression, self-efficacy, and social support [14]. Furthermore, evidence on the effects of pre- and/or postoperative exercise training on HRQoL has been limited to reviews that only included preoperative assessments [28,30], have not provided information on important health dimensions such as global quality of life and cancer-specific symptoms such as fatigue [34], and included non-randomized control trials in the quantitative synthesis [27], which tend to result in larger effect estimates [42].

The first aim of this systematic review was to assess whether exercise training, conducted before and/or after surgery, is an effective intervention to improve postoperative HRQoL in patients with CRC and LC. The second aim was to investigate the effect of exercise training on patients’ fatigue, as this is a highly prevalent symptom after surgery, affecting negatively the HRQoL [4,6,38,39,43].

## 2. Materials and Methods

### 2.1. Protocol and Reporting

The present systematic review was performed according to the Preferred Reporting Items for Systematic Reviews and Meta-Analysis (PRISMA) [44]. The PRISMA checklist is provided in Table S1.

The protocol was pre-registered on the International Prospective Register of Systematic Reviews (PROSPERO), registration number CRD42021246953.

### 2.2. Eligibility Criteria

The eligibility criteria were developed based on the Participants, Intervention, Comparator, Outcome and Type of study (PICOS) approach [45], specific for our review question: In colorectal and lung cancer patients undergoing surgical treatment (P), is pre- and/or postoperative exercise training (I) more beneficial than usual care (C) to improve postoperative HRQoL and fatigue (O)? Only randomized controlled trials (S) were considered.

A detailed description of the eligibility criteria, based on the PICOS approach, is provided below.

#### 2.2.1. Types of Studies

This systematic review included only randomized controlled trials (RCTs) published in English until 21 March 2021. The trials had to allocate participants to a pre- and/or postoperative exercise training intervention versus a control group. Unpublished manuscripts, conference abstracts and systematic reviews were excluded.

#### 2.2.2. Type of Participants

Studies conducted in patients with CRC or LC, awaiting or following surgical resection, were included. Studies recruiting patients with other cancer types were included if data on HRQoL or fatigue were provided for the subgroup of patients with CRC and LC. Patients receiving oncological treatment before or after surgery were included (e.g., chemotherapy and/or radiotherapy).

#### 2.2.3. Type of Intervention

Exercise training, started preoperatively or in the early phase after surgery, i.e., within three months of CRC or LC resection, because this is the period when the most substantial deterioration in HRQoL has been documented [4,5,6,7,8,9]. For research purposes, exercise training was defined as a type of physical activity that consists of a well-defined and structured plan aiming to increase or maintain the person’s physical fitness [46]. Training sessions could be supervised or unsupervised, hospital- or home-based, or a combination of both. Studies that investigated the effectiveness of inspiratory muscle training alone were also included. If exercise training was combined with nutritional or psychological interventions, studies were excluded, because these interventions have also shown beneficial effects on HRQoL [47] and fatigue [16] in cancer patients and could represent a potential confounding factor.

#### 2.2.4. Type of Comparison

The control group could not have performed any type of structured exercise training in the first 3 months after CRC or LC surgery (only usual care with no exercise training). General advice about physical activity, without a structured exercise prescription, was included as a comparison intervention.

#### 2.2.5. Type of Outcome

Primary outcome: HRQoL, measured using a patient-reported outcome measure, after the end of the exercise training program. The HRQoL measures could be generic or cancer-specific.

Secondary outcome: Fatigue, measured after the end of the exercise training program, by a patient-reported outcome measure. 

In the case of preoperative exercise programs, the first assessment of HRQoL or fatigue after surgery was considered. Studies that only reported preoperative values of HRQoL or fatigue were excluded.

### 2.3. Information Sources

A systematic electronic search was carried out in the Physiotherapy Evidence Database (PEDro), PubMed, Scopus, Web of Science and SPORTDiscus, from inception to 21 March 2021. References from retrieved articles were reviewed for additional studies.

### 2.4. Search Strategy

The search strategy combined Key Medical Subject Headings (MeSH) and free-text words related to “colorectal cancer”, “lung cancer”, “surgery”, “exercise training”, “health-related quality of life”, and “fatigue”, using Boolean operators (OR/AND). The full search strategies and filters applied to each bibliographic database are presented in Table S2.

### 2.5. Selection of Studies

The selection of studies started with an independent screening of titles and abstracts by two independent reviewers (PM and SP). If there were doubts about a potential article following the inclusion criteria or if there was incomplete information to make a clear inclusion or exclusion decision, that article was kept for the following phase (analysis of its full text). The second screening phase was also carried out independently by the same reviewers. Studies that were identified by mutual consent were included in the systematic review. In case of disagreement, a third reviewer (JC) was consulted and the final decision was based on the combination of the three opinions. A record of the excluded articles as well as the reasons for their exclusion was kept.

The Cohen’s kappa coefficient was calculated to evaluate interrater reliability in the full text screening [48]. The kappa values can be interpreted as follows: values ≤ 0 as indicating no agreement and 0.01–0.20 as none to slight, 0.21–0.40 as fair, 0.41–0.60 as moderate, 0.61–0.80 as substantial, and 0.81–1.00 as almost perfect agreement [48].

### 2.6. Data Extraction

Data extraction was independently performed by two reviewers (PM and SP) with any discrepancies being resolved through discussion with a third reviewer (JC). Relevant extracted data were organized using standardized tables, that included the following topics: (1) Study characteristics; (2) Participants’ demographic and clinical characteristics; (3) Exercise training dose based on the FITT principles (frequency, intensity, time, and type) [15] and adverse events during exercise interventions; (4) HRQoL/fatigue measures and results. When information regarding any of the above topics was unclear, the authors of the papers were contacted to provide details.

### 2.7. Quality Assessment

Methodological quality of the included studies was assessed by two reviewers (PM and SP), using the Physiotherapy Evidence Database scale (PEDro scale) [49]. Any discrepancies in judgements were resolved by consensus, with a third reviewer (JC) acting as a mediator if necessary. The PEDro scale comprises 11 items: Eligibility criteria, randomized allocation, hidden allocation, baseline comparison between groups, participants, physiotherapists and blind assessors, adequate follow-up, intention to treat the analysis, comparison between groups and point estimate and variability. Based on these items, a score of 0 to 10 is attributed to the RCTs [50]. According to the PEDro scale, studies with a score of 0 to 3 have a “poor” methodological quality, between 4 to 5 “reasonable”, 6 to 8 “good” and 9 to 10 “excellent” [50].

### 2.8. Data Synthesis and Analysis

Meta-analyses were conducted if data from at least three studies or 100 patients could be combined, using standardized mean differences (SMDs) and 95% confidence interval (CI), to allow comparison of data from different instruments [51]. A random-effects model was used in the meta-analysis, as it combines sampling error and between-study variance to estimate effect size [52]. The following thresholds were used to interpret the effect sizes: <0.2 = trivial effect; 0.2–0.5 = small effect; 0.5–0.8 = moderate effect; >0.8 = large effect [53].

The statistical heterogeneity among studies was assessed using the I-squared (*I*^2^), that represents the percentage of variation across studies that is attributable to heterogeneity rather than chance [54]. We adopted the following thresholds: *I*^2^ = 25%: low heterogeneity; *I*^2^ = 50%: moderate heterogeneity; *I*^2^ = 75%: high heterogeneity [54]. If substantial statistical heterogeneity was detected, sensitivity analysis was undertaken by pooling the data of high-quality studies only (PEDro score ≥ 6). When a HRQoL domain was assessed by generic and cancer-specific questionnaires, we performed a subgroup analysis to examine if the exercise training effect was influenced by the type of instrument used.

When not enough data was provided in a study to estimate the exercise training effect, we contacted the authors to provide the required data (mean and standard deviation (SD)). When the post-intervention SD was not reported in a study and not provided by the authors, the pre-intervention SD was used.

All statistical analyses were conducted using the statistical software Comprehensive Meta-Analysis (CMA) (Biostat, Englewood, NJ, USA, version 3.3.070) [55]. A *p*-value of < 0.05 was considered statistically significant.

### 2.9. Publication Bias

The publication bias was calculated using the software CMA [55], generating a funnel plot by the standard error (SE) and the standard difference in means to determine whether the plot was balanced. The risk of publication bias was assessed by the visual inspection of the funnel plots and using Egger’s test to provide a more objective and accurate assessment of funnel plot asymmetry than subjective visual assessment [56].

## 3. Results

### 3.1. Search Results

A total of 1208 records were obtained from electronic databases. After duplicates removal, 1067 records were screened for content, from which 12 RCTs involving 777 patients were included [24,57,58,59,60,61,62,63,64,65,66,67].

**Figure 1:**
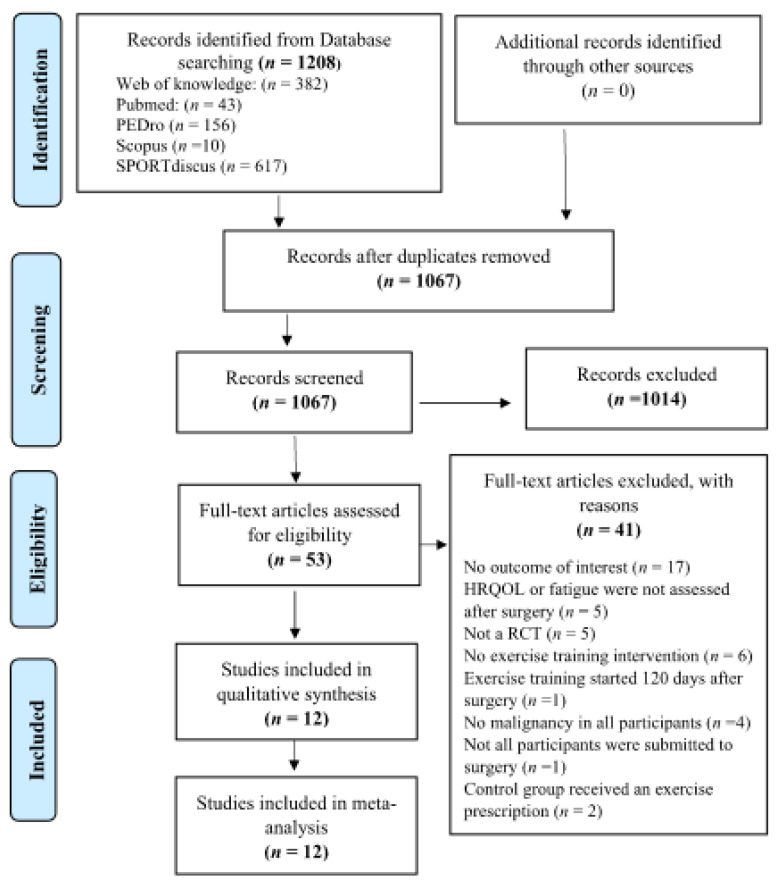
PRISMA flowchart of process of identification of eligible studies; HRQOL (Health-related quality of life); RCT (randomized controlled trial).

The kappa statistics of the agreement between the independent reviewers’ screening of the full-text was 0.87, showing a strong agreement. The flowchart of the literature search, screening and selection process is presented in Figure 1.

### 3.2. Study Characteristics

A total of ten studies included patients with LC (*n* = 651) [24,57,58,60,62,63,64,66,67] and two studies included CRC patients (*n* = 126) [59,65]. Two studies involving LC patients reported results of the same exercise intervention, but with different data [63,66]. One study presented the effects of exercise training on fatigue symptoms 14 weeks after LC surgery [63], and the other provided additional information on HRQoL [66].

The mean age of LC participants was 65.8 years (ranging from 62.6 to 70.9 years), and the proportion of men was 52.8%. All patients had non-small cell lung cancer, the majority with stages I-II (*n* = 454) [24,58,60,61,62,63,67]. A total of 441 patients underwent open thoracotomy and 207 underwent VATS, with unknown surgical approach in three patients. Seven studies reported the extent of surgical resection, which was lobectomy in the majority of participants (*n* = 414) [24,60,61,62,63,64,67]. Four studies reported administration of adjuvant treatment after LC surgery, mainly chemotherapy (*n* = 140) [60,63,64,67]. In one study conducted in the preoperative phase, exclusion criteria included administration of neoadjuvant therapy (chemo- or radiotherapy) prior to surgery [57].

Control groups received usual care without exercise training, consisting in routine physiotherapy treatments plus airway clearance techniques [24,58], routine outpatient appointments after discharge plus pain medication [64], and no advice about exercise training [57,60]. In addition to usual care, the control groups received general instructions on daily activities and weekly phone calls [67], a pedometer with instructions on how to record the total number of steps per day [61], a postoperative exercise intervention initiated 14 weeks after surgery (late exercise intervention) [63,66], and advice to perform physical activity [62]. Two studies reported the levels of physical activity, with no differences between exercise and control groups in light intensity activity and moderate-to-vigorous intensity activity [58,67].

Regarding CRC, the mean age of participants was 59.3 years (ranging from 58.1 to 61.1 years), and the proportion of men was 59.5%. A total of 104 patients had colon cancer (82.5%) and 22 patients had rectal cancer (17.5%), with 18 patients diagnosed with metastatic disease (14.3%) [59,65]. Surgical procedures were reported in one trial and included open surgery (*n* = 18), laparoscopic surgery (*n* = 13) and unknow procedure (*n* = 2) [65]. One study reported that 19.4% of patients (*n* = 18) were submitted to a colostomy surgery [59]. Administration of adjuvant treatment was reported in both studies, mainly chemotherapy (*n* = 75) [59,65] or chemotherapy plus radiotherapy (*n* = 35) [59].

Control groups received usual care, that consisted in no exercise training and instructions for the continuation of usual activities [59,65]. Levels of physical activity were measured in both studies conducted in CRC patients [59,65]. One study reported that at the of the exercise intervention, 88% of the participants in the exercise group performed at least 210 min of moderate to vigorous activity per week, in contrast with 56% in the control group [65]. In the other study [59], the authors reported a significant problem with exercise contamination, because both the exercise and control groups increased their levels of moderate to vigorous activity per week, with no appreciable differences between the two groups. At the end of the intervention, 76% of the participants in the exercise group and 51.6% of the participants in the control group reported more than 60 min of moderate to vigorous activity per week [59]. Table 1 describes the characteristics of the included studies.

### 3.3. Intervention Characteristics

One study implemented the exercise training program before surgery (LC, *n* = 22) [57]. The remaining studies implemented exercise training in the postoperative phase (*n* = 755), starting between the first postoperative day [24] and 73 days after surgery [59].

The exercise training sessions were performed with on-site supervision in eight studies, that were conducted at the hospital [57,62,64,65,67], in a rehabilitation center [63,66] and in a fitness center [60]. In two studies, the exercise program was initiated at the hospital for one week with on-site supervision (inpatient sessions), and continued at home for a period of 12–20 weeks [24,58], with three home visits [24] or weekly telephone supervision [58] by the research staff. In the remaining two studies, the exercise program was performed at home, with a weekly telephone supervision [61,64].

With respect to CRC patients, in one study (*n* = 93) [59] the exercise intervention consisted of aerobic exercise performed at home, with patients being allowed to choose the mode of exercise they preferred (e.g., swimming, cycling or walking). The frequency of aerobic exercise was three to five times per week, 20–30 min per session, at an intensity of 65%–75% of predicted maximal heart rate, during 16 weeks [59]. In the other trial conducted in CRC (*n* = 33) [65], each exercise session was supervised by a physiotherapist, and the exercise training consisted of a combination of resistance exercise (1–2 sets of 10–20 repetitions, 45–75% 1-maximum repetition [1-RM] for all major muscle groups) and moderate-to-high intensity aerobic exercise (cycle-ergometer, alternating intervals at the first ventilatory threshold by three sets of 2 min, with lower intensity intervals by three sets of 4 min), 60 min per session, two sessions per week, over 18 weeks [65].

In LC patients, the total duration of the exercise interventions was approximately 4 weeks in the prehabilitation program (three to five sessions per week, an average of 16 sessions) [57]. In the postoperative phase, the duration of the exercise programs varied from 6 to 20 weeks, with two or three exercise sessions per week in the facility-based programs [60,62,63,64,66,67] and five sessions per week in the home-based program [61].

The most prescribed type of exercise was aerobic training, which was present in all studies, with the exercise mode consisting of cycling in a cycle-ergometer [57,62,63,64,66], walking [24,61] and a combination of cycling and walking [58,67]. The duration of aerobic exercise was reported in six studies and varied from 25 to 30 min [57,61,62,63,66,67]. With respect to the exercise intensity, five studies integrated high-intensity interval training (HIIT), alternating low intensity intervals at 50–60% of peak workload with higher intensity intervals at 80% of peak workload or 85–100% of the maximal heart rate measured by a cardiopulmonary exercise test [57,60,63,66,67]. Six studies included aerobic continuous training with light intensity in the home-based program [61] and moderate-to-high intensity in the hospital-based exercise programs [24,58,62,64].

Nine studies included resistance training, with a volume of two to three sets of 5–15 repetitions [24,57,58,60,62,63,64,66]. The intensity of the resistance training varied between studies, being prescribed a constant load of 0.5–2 kg [62,67], an intensity of 60–80% of one-maximum repetition (1-RM) [63,66], 6–12 RM [60] or an intensity corresponding to a moderate rate of perceived exhaustion according to the OMNI Resistance Scale [57].

In two studies, respiratory muscle training was prescribed [60,62], with a dose of five sets of 10 repetitions followed by 1–2 min of unloaded recovery breathing at 50% of maximal inspiratory and expiratory pressures, using a respiratory muscle trainer device at a rate of 15–20 breaths/min [62].

Four studies (*n* = 164) reported no adverse events of exercise training [57,61,62,65] and one study (*n* = 63) reported a serious adverse event (hip fracture) during balance exercise [60]. A detailed description of the exercise training interventions is presented in Table 2.

### 3.4. Methodological Quality Assessment

The quality assessment showed a mean PEDro score of 6.3, indicating a good methodological quality. No studies were excluded based on methodological quality.

Individually, two RCTs showed a reasonable methodological quality (PEDro score of 5 points) [61,64] and 10 studies a good methodological quality (PEDro scores of 6 and 7 points) [24,57,58,59,60,62,63,65,66,67]. In five studies (42%), a hidden allocation was not performed [59,61,63,64,66]. Owing to the nature of the interventions, blinding of participants and therapists was not performed in any of the included studies. In five studies (42%) outcome assessors were not blinded to group allocation [58,60,61,64,65]. In three studies (25%), intention to treat analysis was not performed [24,57,62]. The methodological quality of the included studies is presented in Table 3.

### 3.5. Synthesis of the Results

A total of 10 studies (*n* = 651) [24,57,58,60,61,62,63,64,66,67] were pooled to assess the efficacy of exercise training on HRQoL and fatigue in patients undergoing LC surgery.

A total of two studies (*n* = 126) [59,65] were pooled to assess the efficacy of exercise training on HRQoL and fatigue in patients undergoing CRC surgery.

#### 3.5.1. Lung Cancer Surgery: Effect of Exercise Training on HRQoL

**Figure 2:**
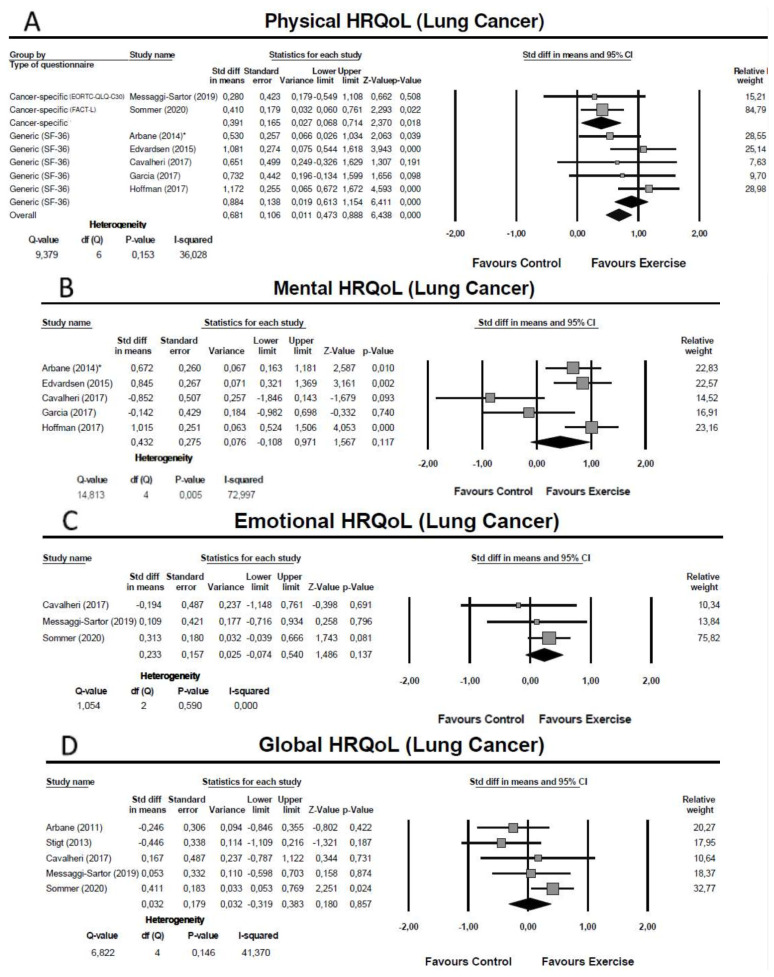
Meta-analysis for the effect estimate of exercise training in lung cancer patients: (**A**) Physical domain; (**B**) Mental Domain; (**C**) Emotional Domain; (**D**) Global health-related quality of life. * Subgroup of patients with airway obstruction (defined as FEV1/forced vital capacity < 0.7).

One study conducted preoperatively [57] and six studies conducted in the postoperative phase (initiated between the first postoperative day and 10 weeks after surgery) [24,60,61,62,66,67] assessed the physical domain of HRQoL Five studies used the physical component summary of a generic questionnaire—the Medical Outcomes Study Short Form 36 General Health Survey (SF-36) [57,58,60,61,67], and two studies used cancer specific-questionnaires–the European Organization for Research and Treatment of Cancer Quality of Life Questionnaire core 30 (EORTC-QLQ-C30) [62] and the Functional Assessment of Cancer Therapy-Lung (FACT-L) [66]. The meta-analysis showed an overall moderate effect of exercise training on the physical domain of HRQoL (SMD = 0.68: 95% CI: [0.47; 0.89]; Z = 6.44; *p* < 0.01; *I*^2^ = 36%) (Figure 2A). Subgroup analysis revealed that the improvement in the physical domain was small when measured using cancer-specific questionnaires (SMD = 0.39: 95% CI: [0.07; 0.71]; Z = 2.37; *p* = 0.02; *I*^2^ = 0%) and large when measured by the SF-36 (SMD = 0.88: 95% CI: [0.61; 1.15]; Z = 6.41; *p* < 0.01; *I*^2^ = 0%) (Figure 2A).

One study conducted preoperatively [57] and four studies conducted in the postoperative phase (initiated between the first postoperative day and 10 weeks after surgery) [58,60,61,67] assessed the mental domain of HRQoL using the mental component summary of the SF-36 questionnaire. The meta-analysis showed no significant effect of exercise training in the mental domain and evidence of substantial heterogeneity (SMD = 0.43: 95% CI: [−0.11; 0.97]; Z = 1.57; *p* = 0.12; *I*^2^ = 73%) (Figure 2B). To explore possible causes of heterogeneity, we undertook a sensitivity analysis including only the studies with good methodological quality [57,58,60,67] but the non-significant effect and the substantial heterogeneity were maintained (SMD = 0.24: 95% CI: [−0.42; 0.90]; *p* = 0.48; *I* = 74%) (see Figure S1).

Three studies conducted postoperatively (initiated between 14 days and 10 weeks after surgery), assessed the emotional domain of HRQoL using the EORTC-QLQ-C30 [62] and the FACT-L [66,67]. The meta-analysis showed no significant effect of exercise training in the emotional domain (SMD = 0.23: 95% CI: [−0.07; 0.54]; Z = 1.49; *p* = 0.14; *I*^2^ = 0%) (Figure 2C).

Five studies conducted postoperatively (initiated between the first postoperative day and 10 weeks after surgery) assessed the global HRQoL using as summary measure the total score of the FACT-L [61], the global quality of life of the EORTC-QLQ-C30 [24,62,67], and the total score of the Saint-George Respiratory Questionnaire (SGRQ) [64]. The meta-analysis showed no significant effect of exercise training in the global HRQoL (SMD = 0.03: 95% CI: [−0.32; 0.38]; Z = 0.18; *p* = 0.86; *I*^2^ = 41%) (Figure 2D).

#### 3.5.2. Lung Cancer Surgery: Effect of Exercise Training on Fatigue

**Figure 3:**
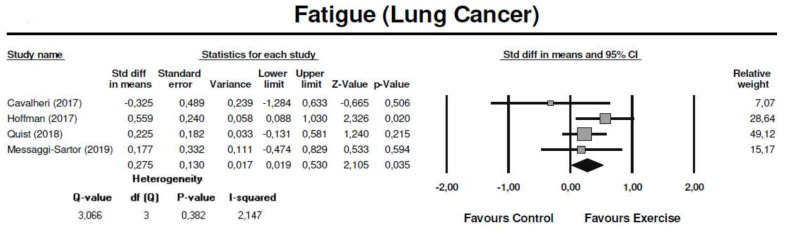
Meta-analysis for the effect estimate of exercise training in lung cancer patients: Fatigue symptoms.

Four studies conducted postoperatively (initiated between 14 days and 10 weeks after surgery) assessed fatigue using the EORTC-QLQ-C30 [62,63], the Functional Assessment of Chronic Illness Therapy-Fatigue subscale (FACIT-Fatigue) [67] and the Brief Fatigue Inventory (BFI) [61] questionnaires. The meta-analysis showed that exercise training significantly reduced fatigue symptoms, with a small effect (SMD = 0.28: 95% CI: [0.02; 0.53]; Z = 2.11; *p* = 0.04; *I*^2^ = 2%) (Figure 3).

#### 3.5.3. Colorectal Cancer Surgery: Effect of Exercise Training on HRQoL

**Figure 4:**
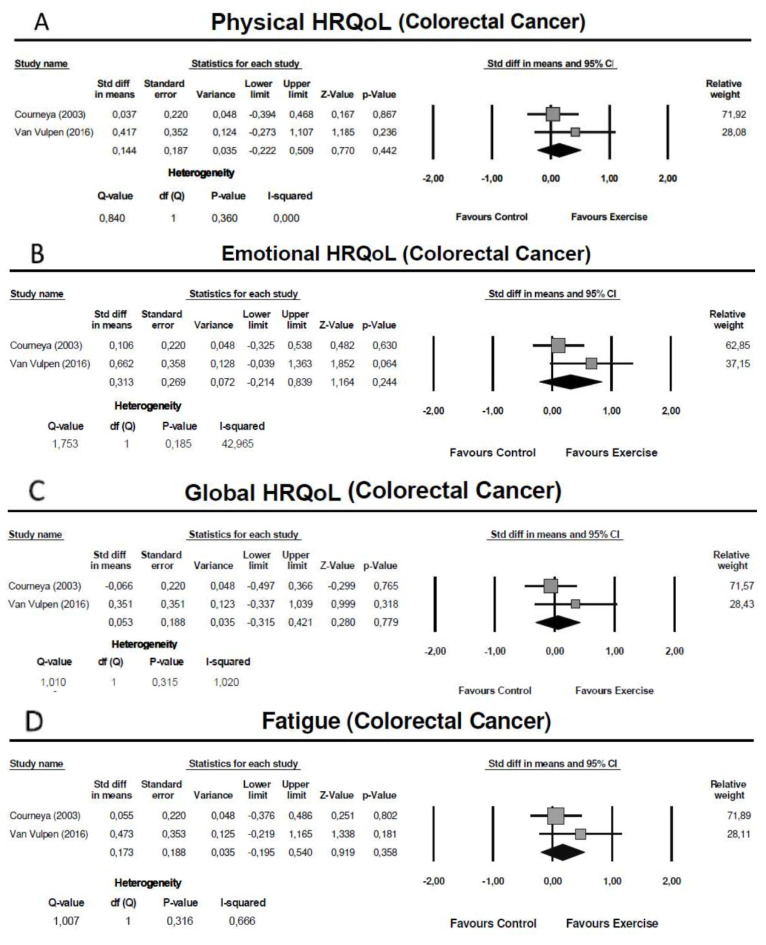
Meta-analysis for the effect estimate of exercise training in colorectal cancer patients: (**A**) Physical domain; (**B**) Emotional Domain; (**C**) Global health-related quality of life; (**D**) Fatigue.

Two studies conducted postoperatively (initiated in the first 10 weeks after surgery), assessed HRQoL using the Functional Assessment of Cancer Therapy-General (FACT-G) [59] and the EORTC-QLQ-C30 [65]. The meta-analysis showed no significant effect of exercise training in the physical domain (SMD = 0.14: 95% CI: [−0.22; 0.51]; Z = 0.77; *p* = 0.44; *I*^2^ = 0%), emotional domain (SMD = 0.31: 95% CI: [−0.21; 0.84]; Z = 1.16; *p* = 0.78; *I*^2^ = 43%) and global HRQoL (SMD = 0.05: 95% CI: [−0.32; 0.42]; Z = 0.28; *p* = 0.78; *I*^2^ = 1%) (Figure 4 A–C).

#### 3.5.4. Colorectal Cancer Surgery: Effect of Exercise Training on Fatigue

Two studies conducted postoperatively (initiated in the first 10 weeks after surgery), assessed fatigue using the Functional Assessment of Cancer Therapy-Fatigue (FACT-F) [59] and the Multidimensional Fatigue Inventory (MFI) [65]. The meta-analysis showed no significant effect of exercise training in fatigue symptoms (SMD = 0.17: 95% CI: [−0.20; 0.54]; Z = 0.92; *p* = 0.36; *I*^2^ = 1%) (Figure 4D).

### 3.6. Publication Bias

For LC, the funnel plot was asymmetrical in the mental domain of HRQoL, indicating the possibility of publication bias. The Egger’s test showed an intercept result of −6.49 (SE = 0.88; 95% CI: [−9.28; −3.71]; *t* = 7.42; *p* = 0.01), confirming strong evidence of publication bias. No evidence of publication bias was found for fatigue and for the physical, mental, emotional and global domains of HRQoL (see Figure S2. For CRC, due to the limited number of included studies, we were not able to generate funnel plots.

## 4. Discussion

This meta-analysis aimed to investigate the effect of exercise training on HRQoL and fatigue after CRC and LC surgery. An improvement was found in the physical domain of HRQoL and in fatigue symptoms after LC resection, with a moderate and small effect, respectively. No evidence was found on the effects of exercise training in HRQoL and fatigue after CRC surgery.

Regarding LC, our results are in agreement with a previous meta-analysis that included exercise interventions undertaken in the first 12 months after surgery, which found beneficial effects in the physical domain of HRQoL, and no evidence of exercise-induced improvements in the mental component and global quality of life [68]. However, in contrast with that meta-analysis which found no effects of exercise training on fatigue [68], our results showed a significant reduction in fatigue in favor of the exercise groups. These results could be due to the inclusion of two studies in our meta-analysis (283 participants) that implemented the exercise interventions in the first month after surgery [61,63], a period when fatigue levels are more severe [4,5,7]. Therefore, considering that the effect of exercise interventions is greater in cancer patients with higher fatigue levels [69], the early initiation of exercise training after LC resection could be an important factor to mitigate this symptom. This hypothesis is corroborated by a large clinical trial which compared the effect of an exercise intervention initiated 14 days after LC resection in contrast with an intervention initiated at week 14, and found a significant difference in fatigue levels in favor of the early initiated exercise program [63].

Our review thereby contributes to the current literature by providing evidence that exercise interventions initiated preoperatively or in the first 3 months after surgery lead to a significant better physical function (pre- or postoperative exercise training) and reduce fatigue symptoms (postoperative exercise training) when compared to control groups, with no exercise training.

We have also found that the improvements in the physical domain appear to be smaller with cancer-specific questionnaires when compared to a generic instrument (SF-36). This variation in the exercise effect could be partially explained by the low correlation between the physical component summary of the SF-36 and the physical functioning of the EORTC-QLQ-C30, as observed in a prospective analysis of LC patients submitted to surgical resection, suggesting that the two instruments possibly reflet different aspects of the physical domain and may be complementary [70].

The beneficial effects of exercise training in the physical domain of HRQoL could be relevant both to address patients’ needs and in terms of survival, because the deterioration in the physical function is perceived by LC patients as an extremely undesirable consequence of surgery [71], and a 10% decrease in this domain during the first 6 months after LC surgery was associated with 18% higher risk of death [37].

Contrary to the substantial deterioration in the physical function and fatigue, the mental/emotional domains tend to return to preoperative levels or even improve after LC surgery [5,7,11,72], showing less need for exercise interventions. With respect to global HRQoL, the only trial that found significant improvements of exercise training was implemented 14 days after surgery and used an LC specific module [66], reinforcing the importance of start exercise interventions earlier after surgery and choose a specific questionnaire, which is more accurate to detect perioperative changes in LC symptoms [70].

It should be emphasized that only one of the included studies was conducted in the preoperative phase [57], achieving significant improvements in the physical domain of HRQoL three months after LC surgery. These results, together with the findings of a previous meta-analysis showing that higher preoperative levels of physical activity were significantly associated with better HRQoL after oncological surgery [73], emphasize the need for further research to investigate if preoperative exercise training can prevent the detrimental impact of LC resection in HRQoL. This could be particularly relevant for the subgroup of patients receiving neoadjuvant therapy, that were excluded from this study, since neoadjuvant chemotherapy was associated with lower preoperative aerobic capacity and thus impact the short- and long-term outcome of tumor resection [74].

The small number of included studies and the heterogeneity of the exercise training prescribed prevent us from providing recommendations about a specific exercise dose to improve HRQoL and fatigue after LC surgery. Nevertheless, consistent improvements in physical function and fatigue were found in four studies combining HIIT plus resistance exercise [57,60,63,66] all of them presenting good methodological quality. Additionally, all these exercise interventions combining HIIT plus resistance exercise improved patients’ aerobic capacity, a predictor of better prognosis after LC surgery [32,75,76] and a factor associated with better HRQoL in LC patients who previously completed curative intent treatment [22]. As shown in other cancer types, the therapeutic benefits of HIIT on HRQoL and fatigue may be mediated by improvements in aerobic capacity [77] and in a short time frame as the perioperative phase and the prescription of a higher exercise intensity could be a relevant factor to achieve central and peripheral physiological adaptations [78,79,80]. Furthermore, higher exercise intensities appear to protect against chemotherapy-related inflammation [81], a mechanism involved in the pathogenesis of fatigue [82], which could be clinically relevant for patients eligible to adjuvant treatment after surgery. This rationale is supported by a large clinical trial which found that a combination of high-intensity aerobic and resistance exercise was significantly more effective to reduce physical fatigue compared to low-to-moderate-intensity exercise in cancer patients undergoing (neo-)adjuvant treatment [83].

As for CRC, no effects of exercise training in postoperative HRQoL and fatigue were found. However, these results need to be interpreted with prudence because the effect estimates were only based on two clinical trials with inconsistent findings: a supervised intervention combining moderate- to vigorous-intensity aerobic plus resistance exercise achieved beneficial effects in fatigue symptoms and physical function [65], while a home-based intervention incorporating moderate-intensity aerobic exercise found no benefits in these clinical outcomes [59]. The lack of significant results in the home-based exercise intervention [65] may be partially explained by three factors: (1) High contamination rate (51.6%), with the participants in the control group significantly increasing their levels of moderate to vigorous physical activity, a factor associated with enhanced recovery in self-reported physical functioning after CRC surgery [84]; (2) Adherence rates, that were slightly lower than those observed in the supervised intervention (76% vs. 89%); (3) Type of exercise prescribed, since the clinical guidelines in oncology indicates that combining aerobic and resistance training leads to higher benefits in HRQoL compared with programs involving only aerobic or resistance exercise [15], and the efficacy of resistance exercise programs appears to be superior than aerobic exercise to reduce fatigue levels among cancer patients [85], possibly by the attenuation of muscle wasting and disruptions in muscle metabolism caused by chemotherapy, such as oxaliplatin [82].

In contrast with our results, a previous systematic review concluded that exercise training is effective for improving HRQoL and fatigue following a diagnosis of CRC [84]. However, it did not focus on pre- and/or postoperative exercise interventions, including patients undergoing cancer treatment and long-term survivors [84]. Our review adds knowledge to this field of research by underlining the need to conduct further studies to assess the effect of perioperative exercise interventions in these clinical outcomes.

### 4.1. Implications for Future Research

Considering that the most substantial deterioration in HRQoL and fatigue occurs in the early phase after surgery [5,6,7] and based on the positive association between preoperative physical activity levels and postoperative HRQoL [73], future high-quality trials should explore if prehabilitation exercise programs could prevent the deleterious effects CRC and LC surgery in these clinical outcomes.

Future clinical trials should also target patients with lower physical function and higher fatigue levels, the subgroup of individuals that appears to benefit most from exercise interventions [69], and use cancer-specific modules such as the EORTC-QLQ-LC13/CR29, which provides a more detailed evaluation of cancer-specific symptoms in comparison with generic questionnaires [70]. Finally, more research is warranted to identify the optimal exercise dose to improve HRQoL after CRC and LC surgery.

### 4.2. Strengths and Limitations

Strengths of the current review consist of the use of the PRISMA guidelines [85], the extensive search in multiple databases, the independent and robust screening process, the provision of a detailed description of the exercise interventions based on the FITT principles, and a comprehensive quantitative synthesis of the exercise training effects in the different domains of HRQoL.

There are, however, some limitations that need to be acknowledged. First, only two RCTs including CRC patients were eligible for inclusion, preventing us to provide more precise estimates of exercise effects on HRQoL and fatigue after surgery. Second, the majority of patients in the included studies had early-stage disease, being admitted to curative resection. Therefore, the exercise training effects may not be generalized to patients with advanced-stage disease selected for palliative surgery. Third, although the eligible studies had an overall good methodological quality, in five trials a concealed allocation was not carried out [59,61,63,64,66], which could lead to an overestimation of the exercise training effects [86]. Lastly, the possibility of language bias should not be neglected because only studies published in English were considered to inclusion [87].

## 5. Conclusions

The results of our meta-analysis indicate that exercise training is an effective intervention to improve the physical domain of HRQoL and reduce fatigue levels after LC surgery, compared with usual care. Considering that these dimensions are especially affected as a consequence of surgical resection, exercise training could be a relevant supportive intervention to target patients’ needs. Further studies are necessary to clarify the effects of exercise training on HRQoL and fatigue after CRC surgery.

## Figures and Tables

**Table 1:** Characteristics of included studies/participants and key findings.

Reference	Sample Size/Sex/Age (Mean)	Type of Cancer/Stage	Surgical Approach	Timing of Intervention	Exercise Group	Control Group	Primary Outcome	Assessment (HRQoL/ Fatigue)	Key Findings (HRQoL/Fatigue)
Courneya et al., 2003 [59]	E.G (*n* = 62) M = 34; F = 28 59.9 years C.G (*n* = 31) M = 20; F = 11 61.1 years	CRC Stage III-IV (82.4%) Node stage 0 (60%) Metastatic (3.5%)	Not reported (19.4% of patients submitted to colostomy)	Post-surgery (mean of 73 days)	Home-based Aerobic exercise	Usual care (no exercise training)	HRQoL 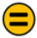	FACT-G and FACT-F	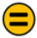 No between-group differences observed in HRQoL and fatigue
Arbane et al., 2011 [24]	E.G. (*n* = 26) Sex not reported 62.6 years C.G. (*n* = 25) Sex not reported: 65.4 years	NSCLC Stage I–IV	VATS (*n* = 2) Open Thoracotomy (*n* = 49)	Post-Surgery (1st postoperative day)	Aerobic exercise + Resistance exercise + Mobility exercises	Usual care (Routine physiotherapy treatments, airway clearance techniques, mobilization and upper limb activities)	Exercise capacity 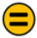	EORTC-QLQ-C30	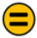 No between-group differences observed in HRQoL
Stigt et al., 2013 [64]	E.G (*n* = 23) M = 21; F = 2 63.6 years C.G (*n* = 26) M = 19; F = 7 63.2 years	NSCLC Stage not reported	Open Thoracotomy (*n* = 49)	Post-surgery (4 weeks after hospital discharge)	Aerobic exercise + Resistance exercise	Usual care (routine outpatient appointments after discharge, pain medication)	HRQoL 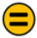	SGRQ	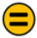 No between-group differences observed in HRQoL
Arbane et al., 2014 [58]	E.G. (*n* = 64) M = 29; F = 35 67 years C.G. (n = 67) M = 43; F = 24 68 years	NSCLC Stage I-IV	VATS (*n* = 38) Open Thoracotomy (*n* = 90) Unknown (*n* = 3)	Post-Surgery (1st postoperative day)	Aerobic exercise + Resistance exercise (In-hospital) + Home-based walking Program	Usual care (Routine physiotherapy treatments, airway clearance techniques, mobilization, and upper limb activities)	Physical activity levels 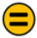	SF-36	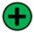 Significant difference between groups in PCS and MCS in patients with airflow obstruction
Edvardsen et al. 2015 [60]	E.G. (*n* = 30) M = 13; F = 17 64.4 years C.G. (*n* = 31) M = 15; F = 16 65.9 years	NSCLC Stage I-IV	VATS (*n* = 10) Open Thoracotomy (*n* = 51)	Post-surgery (5 to 7 weeks after surgery)	HIIT + Resistance exercise + daily inspiratory muscle training	Standard postoperative care (no advice about exercise training)	Exercise capacity 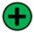	SF-36 and EORTC-QLQ-C30	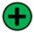 Significant difference between groups in PCS, MCS and dyspnea, favoring the exercise group
Van Vulpen et al., 2016 [65]	E.G. (*n* = 17) M= 10; F= 7 58.1 years C.G. (*n* = 16) M= 11; F= 5 58.1 years	Colon cancer Stage M0	Laparoscopic (*n* = 13) Open surgery (*n* = 18) Unknown (*n* = 2)	Post-surgery (10 weeks after surgery, during adjuvant chemotherapy)	Aerobic exercise (Interval training) + Resistance Exercise	Usual Care (no exercise intervention and instruction for the continuation of the usual activities)	Fatigue 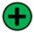	EORTC-QLQ-C30, MFI	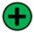 Significant difference between groups in physical function and both physical and general fatigue, favoring the exercise group.
Cavalheri et al., 2017 [67]	E.G. (*n* = 9) M= 3; F= 6 66 years C.G. (*n* = 8) M= 2; F= 6 68 years	NSCLC Stage I-IIIA	VATS (*n* = 9) Open Thoracotomy (*n* = 8)	Post-surgery (6 to 10 weeks after lobectomy or 4 to 8 weeks after last chemotherapy cycle)	Aerobic exercise (Continuous training and HIIT) + Resistance Exercise	General Instructions on daily activities + weekly phone calls	Exercise capacity 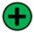	SF-36, EORTC-QLQ-C30, FACT-L and FACIT-F	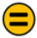 No between-group differences observed in HRQoL and fatigue
Garcia et al., 2017 [57]	E.G (*n* = 10) M = 9; F = 1 70.9 years C.G (*n* = 12) M = 11; F = 1 69.4 years	NSCLC Stage not reported	VATS (*n* = 22)	Pre-surgery (baseline assessment was 54.5 days before surgery)	HIIT + Resistance Exercise + Breathing exercises (incentive spirometer)	Usual care (no exercise training)	Exercise capacity 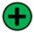	SF-36	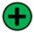 Significant difference between groups in PCS, favoring the exercise group
Hoffman et al., 2017 [61]	E.G (*n* = 37) M = 17; F = 20 64.4 years C.G (*n* = 35) M = 15; F = 20 65.6 years	NSCLC Stage I-IV	VATS (*n* = 13) Open Thoracotomy (*n* = 59)	Post-surgery (mean of 4 days after hospital discharge)	Home-based aerobic exercise + balance Training	Usual care from health providers plus a pedometer with instructions on how to record the total number of steps per day	Feasibility and acceptability	SF-36 and BFI	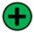 Significant difference between groups in the PCS 3 months after surgery, favoring the preoperative exercise group
Quist et al., 2018 [63] and Sommer et al. 2020 [66]	E.G (*n* = 110) M = 46; F = 64 66 years C.G (*n* = 101) M = 48; F = 53 65 years	NSCLC Stage IA-IIIB	VATS (*n* = 110) Open Thoracotomy (*n* = 101)	Post-surgery (14 days after surgery: early intervention)	HIIT + Resistance exercise + breathing exercises combined with stretching and tension-release techniques	Usual care (postoperative exercise initiated 14 weeks after surgery: late intervention)	Exercise capacity * 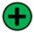	EORTC-QLQ-C30 and FACT-L	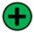 Significant difference between groups in fatigue and HRQoL 14 weeks after surgery favoring the early initiated intervention
Messaggi-Sartor et al., 2019 [62]	E.G (*n* = 16) M = 8; F = 8 64.2 years C.G (*n* = 21) M = 18; F = 3 64.8 years	NSCLC Stage I-II	VATS (*n* = 3) Open Thoracotomy (*n* = 34)	Post-surgery (6–8 weeks after lung resection)	Aerobic exercise + Resistance exercise + Respiratory muscle training	Advice to perform physical activity, following WHO recommendations.	Exercise capacity 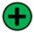	EORTC-QLQ-C30	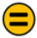 No between-group differences observed in HRQoL

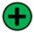
 Significant differences between the exercise group and the control group; 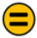 No between-group differences. * Measurement at 14 weeks after surgery (end of the early initiated exercise intervention), BFI (Brief fatigue inventory); C.G (Control Group); CRC (Colorectal cancer); E.G (Exercise Group); EORTC-QLQ-C30 (European Organization for Research and Treatment of Cancer questionnaire); FACIT-F (Functional Assessment of Chronic Illness Therapy—Fatigue); FACT-F (Functional assessment of cancer therapy—Fatigue); FACT-G (Functional assessment of cancer therapy—General); FACT-L (Functional assessment of cancer therapy—Lung); HIIT (High Intensity Interval Training); HRQOL (Health-related quality of life); M (Male); MCS (Mental Component Summary); MFI (Multidimensional Fatigue Inventory); NSCLC (Non-small cell lung cancer); F (Female); PCS (Physical Component Summary); SF-36 (Short-Form 36-Item Health Survey; VATS (Video-assisted thoracoscopic surgery); WHO (Word Health Organization).

**Table 2:** Characteristics of the exercise training interventions (12 studies).

Reference	Type of Supervision	Type/Mode of Exercise	Time (min/sets)/Intensity (MET; % HR_max_; %HRR;% W_peak_; % 1- RM)	Progression (Time/Sets/Intensity)	Session Time (min)	Frequency (Sessions Per Week)	Program Duration (Weeks/Sessions)	Adverse Events (A.E) Attrition/Adhrence Rates
**Aerobic Exercise**								
Courneya et al., 2003 [59]	Telephone (weekly telephone calls)	Aerobic: Swimming, cycling or walking	Aerobic: 20–30 min 65–75% of predicted HR_max_.	Aerobic: Varied depending on motivation and capability	20–30 min	3–5 times per week	16 weeks	A.E: NR Adherence: 76% Attrition: 10%
Hoffman et al., 2017 [61]	Mixed supervision: presential (two home visits) and by telephone	Aerobic: Continuous walking in place with Wii Fit Plus exercise equipment Balance exercises: Wii balance exercises	Aerobic: 5 min each day for 5 days during week 1 Light intensity (less than 3.0 METs)	Aerobic: The walking time was increased by 5 min each week with the goal of 30 min per day during week 6	5 min (1st week) to 30 min (6th week)	5 times per week	6 weeks	A.E: No adverse events Adhrence:93% Attrition:3%
**Aerobic + Resistance Exercise**								
Arbane et al., 2011 [24]	Presential in hospital and three home visits during the home-based program	Aerobic: Walking Resistance: Seated leg raises and unspecified home resistance exercises	Aerobic: 60% to 80% HRmax (220-age formula) Resistance (in-hospital): Ankle weights of 2 lb.	Resistance (in-hospital): Progress to ankle weights of 4 lb	NR	In hospital: 5 times per week/twice daily Home-based: NR	In-Hospital: 5 days Home-based: 12 weeks	A.E: NR Adherence: NR Attrition: 17%
Stigt et al., 2013 [64]	Presential	Aerobic: Continuous training on a cyclo-ergometer Resistance: Dose not reported	Aerobic: 60% to 80% of peak workload	NR	60 min	2 times per week	12 weeks	A.E: NR Adherence: NR Attrition:26.5%
Arbane et al., 2014 [58]	Mixed supervision: Presential (In hospital program) and weekly telephone calls (home program)	Aerobic: Cycling (in hospital) and home-based walking program (SPACE) Resistance: NR	Aerobic: 30 min 60% to 90% of HRR 3 or 4 on the Borg CR10 13 to 15 on the Borg RPE Pedalling rate was held between 50 and 60 rpm Resistance: 10-RM (number of. Sets NR)	NR	NR	NR	In Hospital: 5 days Home-based: 20 weeks	A.E: NR Adherence: NR Attrition: 31%
Van Vulpen et al., 2016 [65]	Presential (In-hospital)	Aerobic: Interval training on a cycle-ergometer Resistance: Exercises for major muscle groups (arms, legs, shoulder and trunk)	Aerobic High intensity: 3 sets x 2 min at the first VT measured by CPET Low intensity: 3 sets x 4 min below the first VT Resistance: 2 sets x 10 reps at 65% of 1-RM	Aerobic High intensity: 2 sets x 7 min at the first VT Low intensity: 1 set x 7 min at the first VT Resistance: 1 set x 10 reps (75% 1-RM) and 2 sets x 20 reps (45% 1-RM)	60 min	2 sessions per week	18 weeks	A.E: No serious adverse events Adherence: 89% Attrition:15%
Cavalheri et al., 2017 [67]	Presential (In-hospital)	Aerobic: Continuous walking on treadmill or 100-m corridor + HIIT on a cycle-ergometer Resistance: Exercises for upper and lower limbs using hand weights (step-ups, elbow flexion and shoulder abduction)	Walking on corridor—20 min at 80% of the average 6MWT speed Treadmill walking—20 min at 70% of the average 6MWT speed Cycling (HIIT): 14 min High intensity (2 × 2 min at 80% of the peak workload) Low intensity (10 min at 60% of the peak workload) Resistance training: 2–3 sets of 10 reps; Initial hand weights: 1.5 kg for women and 2 kg for men	Aerobic: Walking speed was increased if the patients were able to walk for 20 min continuously providing symptoms and SpO_2_ were within acceptable limits (≥88%)	60 min	3 sessions per week	8 weeks	A.E: NR Adherence: 44% Attrition: 0%
Garcia et al., 2017 [57]	Presential (In-Hospital)	Aerobic: HIIT on a cycle-ergometer Resistance: 6 exercises using elastic bands and body weight, targeting the main muscle groups.	Aerobic: 30 min High intensity (1 min at 80% of peak workload) Low intensity (4 min at 50% of peak workload) Resistance: 3 sets of 15 reps (45” rest) Moderate perceived rate of exhaustion (OMNI Scale)	Aerobic: Peak workload adjusted based on an incremental cycle test (10th session) Resistance: Number of sets was increased to four if tolerated (10th session)	60 min	3–5 times per week depending on the surgery date	16 sessions (range 8–25)	A.E: No adverse Events Adherence: NR Attrition: 14%
Quist et al., 2018 [63] and Sommer et al. [66]	Presential (Rehabilitation center)	Aerobic: HIIT on a cycle-ergometer Resistance: 5 exercises using weight machines (leg press, chest press, leg extension, pull to chest, and pull down)	Aerobic: 25 min High intensity (1–2 min at 85%–100% of HR_max_) Low intensity: (1-min pauses) Resistance: 3 sets of 5 to12 reps at 60–80% of 1-RM	Aerobic: 50–60% of HR_max_. (first 4 weeks) and 70–90% of HR_max_ (last 8 weeks) Resistance: Every two weeks, the load was increased and the number of repetitions reduced to a final of 3 sets of 8 reps	60 min	2 times per week	12 weeks (24 sessions on nonconsecutive days)	A.E: NR Adherence: NR Attrition: 40%
**Aerobic + Resistance + Respiratory Muscle Exercise**								
Edvardsen et al., 2015 [60]	Presential (Fitness center)	Aerobic: HIIT (walking uphill on treadmill) Resistance: Leg press, leg extension, back extension, seat row, bicep curls, chest-and-shoulder press Inspiratory muscle training	Aerobic: HIIT achieving 80–95% of the HR_max_. Resistance training: 3 sets of 6–12 RM Inspiratory muscle training: NR	Aerobic: The intensity and duration of each interval was individually increased based on the patient’s improvement and symptoms of fatigue and dyspnoea	60 min	3 times per week	20 weeks	A.E: Hip fracture during balance training Adherence: 88% Attrition: 12%
Messaggi-Sartor et al., 2019 [62]	Presential (In-Hospital)	Aerobic: Continuous training on a cycle-ergometer IEMT: Using a respiratory muscle trainer at a rate of 15–20 breaths/min Resistance: 3 exercises (bicep curl, chest and shoulder press	Aerobic: 30 min 60% of peak workload IEMT: 5 sets of 10 repetitions followed by 1–2 min of unloaded recovery breathing (off the device) 50% of PI_max_ and PE_max_; Resistance: 3 sets with a constant load of 0.5 kg.	Aerobic: Increased in workload by 5 watts weekly if the patient was able to tolerate the set load for 30 min IEMT: Load was adjusted weekly by 10 cmH_2_O if tolerated	60 min	3 times per week (IEMT: twice a day)	8 weeks (24 sessions)	A.E: No adverse events Adherence: >80% Attrition: 35%

Borg CR-10 (Borg Category-Ratio 10); HIIT (High intensity interval training); HRR (Heart rate reserve); IEMT (Inspiratory and expiratory muscle training); HR_max_ (Maximal Heart Rate); MET (Metabolic Equivalent of task); RM (Repetition Maximum); reps (repetitions); min (minutes); PI_max_ (Maximal inspiratory pressure); PE_max_ (Maximal expiratory pressure); RPE (Rate of perceived exertion); VT (ventilatory threshold); 6MWT (6 min walk test).

**Table 3:** Methodological quality assessment using the PEDro scale (12 studies).

Reference	Eligibility Criteria *	Randomized Allocation	Hidden Allocation	Baseline Comparison Between Groups	Blind Participants	Blind Physical Therapists	Blind Assessors	Proper Follow-Up	Intention to Treat Analysis	Comparison Between Groups	Point Estimate and Variability	Total Score
Courneya et al., 2003 [59]	√	√	×	√	×	×	√	√	√	√	√	7/10
Arbane et al., 2011 [24]	√	√	√	√	×	×	√	√	×	√	√	7/10
Stigt et al., 2013 [64]	√	√	×	√	×	×	×	×	√	√	√	5/10
Arbane et al., 2014 [58]	√	√	√	√	×	×	×	×	√	√	√	6/10
Edvardsen et al. 2015 [60]	√	√	√	√	×	×	×	√	√	√	√	7/10
Van Vulpen et al., 2016 [65]	√	√	√	√	×	×	×	√	√	√	√	7/10
Cavalheri et al., 2017 [67]	√	√	√	√	×	×	√	×	√	√	√	7/10
Garcia et al., 2017 [57]	√	√	√	√	×	×	√	×	×	√	√	6/10
Hoffman et al., 2017 [61]	√	√	×	√	×	×	×	×	√	√	√	5/10
Quist et al., 2018 [63]	√	√	×	√	×	×	√	×	√	√	√	6/10
Messaggi-Sartor et al., 2019 [62]	√	√	√	√	×	×	√	×	×	√	√	6/10
Sommer et al., 2020 [66]	√	√	×	√	×	×	√	×	√	√	√	6/10

* Eligibility criteria item does not contribute to total score.

## Data Availability

The data presented in this study are available on request from the corresponding author.

## Supplementary Materials

The following are available online at https://www.mdpi.com/article/10.3390/cancers13194975/s1, Table S1: Preferred Reporting Items for Systematic Reviews and Meta-Analyses (PRISMA) checklist, Table S2: Database search strategy, Figure S1: Sensitive analysis mental domain of health-related quality of life (Lung Cancer); Figure S2: Publication Bias (Lung Cancer): Physical, Mental, Emotional and Global domains of HRQoL; Fatigue.
